# SeqCAT: Sequence Conversion and Analysis Toolbox

**DOI:** 10.1093/nar/gkae422

**Published:** 2024-05-27

**Authors:** Kevin Kornrumpf, Nadine S Kurz, Klara Drofenik, Lukas Krauß, Carolin Schneider, Raphael Koch, Tim Beißbarth, Jürgen Dönitz

**Affiliations:** Department of Medical Bioinformatics, University Medical Center Göttingen, Goldschmidtstr. 1, 37077 Göttingen, Germany; Department of Medical Bioinformatics, University Medical Center Göttingen, Goldschmidtstr. 1, 37077 Göttingen, Germany; Göttingen Comprehensive Cancer Center (G-CCC), 37075 Göttingen, Germany; Department of Medical Bioinformatics, University Medical Center Göttingen, Goldschmidtstr. 1, 37077 Göttingen, Germany; Department of General, Visceral and Pediatric Surgery, University Medical Center Göttingen, Robert-Koch Str. 40, 37075 Göttingen, Germany; Department of General, Visceral and Pediatric Surgery, University Medical Center Göttingen, Robert-Koch Str. 40, 37075 Göttingen, Germany; Department of Hematology and Medical Oncology, University Medical Center Göttingen, Robert-Koch Str. 40, 37075 Göttingen, Germany; Department of Medical Bioinformatics, University Medical Center Göttingen, Goldschmidtstr. 1, 37077 Göttingen, Germany; Campus Institute Data Science (CIDAS), Göttingen, Germany; Department of Medical Bioinformatics, University Medical Center Göttingen, Goldschmidtstr. 1, 37077 Göttingen, Germany; Göttingen Comprehensive Cancer Center (G-CCC), 37075 Göttingen, Germany; Campus Institute Data Science (CIDAS), Göttingen, Germany

## Abstract

Dealing with sequence coordinates in different formats and reference genomes is challenging in genetic research. This complexity arises from the need to convert and harmonize datasets of different sources using alternating nomenclatures. Since manual processing is time-consuming and requires specialized knowledge, the Sequence Conversion and Analysis Toolbox (SeqCAT) was developed for daily work with genetic datasets. Our tool provides a range of functions designed to standardize and convert gene variant coordinates based on various sequence types. Its user-friendly web interface provides easy access to all functionalities, while the Application Programming Interface (API) enables automation within pipelines. SeqCAT provides access to human genomic, protein and transcript data, utilizing various data resources and packages and extending them with its own unique features. The platform covers a wide range of genetic research needs with its 14 different applications and 3 info points, including search for transcript and gene information, transition between reference genomes, variant mapping, and genetic event review. Notable examples are ‘Convert Protein to DNA Position’ for translation of amino acid changes into genomic single nucleotide variants, or ‘Fusion Check’ for frameshift determination in gene fusions. SeqCAT is an excellent resource for converting sequence coordinate data into the required formats and is available at: https://mtb.bioinf.med.uni-goettingen.de/SeqCAT/.

## Graphical Abstract

**Graphical Abstract:**
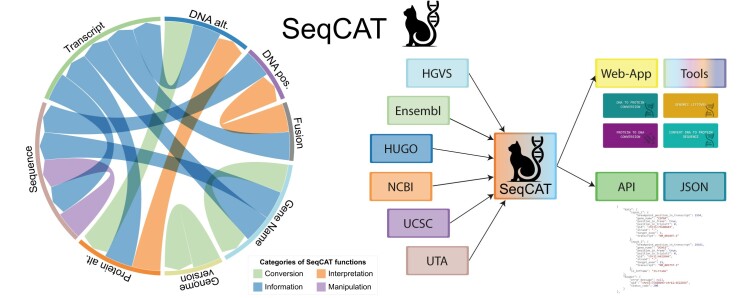

## Introduction

The growth of digital data is transforming biomedical research, particularly propelled by the rapid expansion of sequencing data due to advancements in next-generation sequencing (NGS) over the last decade (1). This has resulted in higher resolution and lower costs, enabling genome-wide association studies (GWAS) (2,3) and the discovery of the molecular basis of various diseases. However, a major challenge is curating data from different genome assemblies to accurately merge datasets from multiple sources.

The possible annotation on different levels, e.g. DNA, transcript or protein, makes it difficult to compare studies and can lead to errors and increased workload. Existing conversion tools or websites often require specific input formats and handling. These minutiae require more time than appropriate and distract from the main task. This can be cumbersome and time-consuming when integrating data from multiple sources for further processing. This holds true for single annotations or harmonizing bulk data for common analysis.

**Figure 1.**
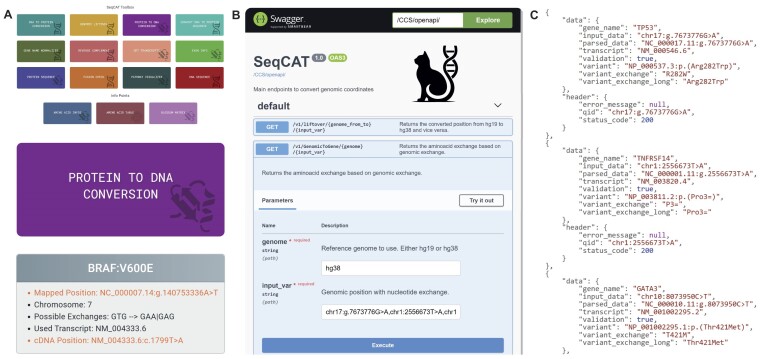
Overview of the SeqCAT toolbox. (**A**) Overview of all current functions. (top) SeqCAT’s frontend toolbox shows several functionalities, e.g. ’Protein to DNA Conversion’, in the form of small colored tiles. (middle). The frontend output is shown in small boxes. The header contains the input, while the content of the box shows the detailed results. Here the ’Protein to DNA Conversion’ shows the translation of *BRAF*: *V*600*E* into the gene position. The *Possible Exchange* line lists all possible triplets (coding strand) of the input alteration, while the *Mapped Position* line shows the automatic selected exchange: *chr*7 : 140753336*A* > *T* (reference genome) (bottom). (**B**) SeqCAT’s SwaggerUI documentation allows visual exploration and provides an overview of all functionalities and a description of how to use the API on the command line or within pipelines and workflows. (**C**) Using SeqCAT’s API returns a standardized, machine-readable JSON format that can be easily integrated into other pipelines. The *header* returns the input identifier (*qid*) and whether a request could be processed (*status_code*: 200–success, 400–error). This allows each input to be correctly mapped to its corresponding output in batch processing. The *data* section returns the results processed by SeqCAT.

To address the need for standardized data annotation in the scientific community, the **Seq**uence **C**onversion and **A**nalysis **T**oolbox (SeqCAT) has been developed. SeqCAT is a versatile tool that can annotate, process, and retrieve sequence data information. It currently provides 14 distinct functionalities and 3 info points, covering a wide range of research needs, from the categories conversion, information retrieval, interpretation and manipulation. The user-friendly frontend application simplifies the usability for small data sets and makes complex tasks more accessible. Its robust API (Application Programming Interface) for projects with larger data sets allows for easy integration into existing pipelines and streamlines workflows (Figure 1). SeqCAT is a versatile and adaptable platform for the scientific community, addressing major challenges in sequence data management and analysis.

## Materials and methods

### Packages and frameworks

SeqCAT’s backend is developed in Python v3.9 (4) using packages like Flask v2.1.1 (https://palletsprojects.com/), Pandas v1.4.2 (5) and hgvs v1.5.1 (6). Sequence and transcript data are fetched by the seqrepo repository version ’2021-01-29’ (7) and the UTA-database version ’uta_20210129b’ (6). The frontend application is implemented using the Angular Framework v16.1.4 (https://angular.io/) including a SwaggerUI API documentation. Frontend and backend applications are dockerized through Docker v25.0.4 (8).

### Data and other resources

The HGNC custom download section was used to create a file of approved, alias, and previous gene symbols for gene name normalization (9). Gene names requiring transcript conversion are matched to their corresponding NCBI MANE transcript, obtained from the refseq/mane directory of the NCBI FTP server, version 1.3 (10). Liftover operations use chains obtained from the UCSC FTP server (11). For pathway visualization, data is retrieved from the KEGG PATHWAY database, selecting pathways and gene information using KEGG’s hsa identifier (12).

## Results

SeqCAT is a comprehensive toolbox designed to simplify everyday laboratory tasks, offering a wide range of features to enhance research workflows and integrates seamlessly into analysis pipelines. SeqCAT facilitates tasks such as conversion between genomic and protein sequences and provides access to information on genes, transcripts, pathways and other resources. SeqCAT was initially created to map genomic variants to their corresponding protein changes and vice versa. Within the scope of various projects and growing demands, the toolbox was extended by more functionalities. It has expanded to a comprehensive platform where a collection of useful tools are easily accessible, making it a valuable resource for scientific research.

Key features of SeqCAT include:

- **DNA to protein conversion** maps genomic alterations to their corresponding protein exchanges, based on the MANE Select transcript (Matched Annotation from the NCBI and EMBL-EBI) (10).
- **Protein to DNA conversion** maps protein positions according to the HGVS annotation (13) to all of their possible genomic exchanges, and chooses the exchange with the minimum number of base pair exchanges.
- **Genomic liftover** maps genomic data between common reference genomes.
- **Gene name normalizer** tests HGNC gene symbols for approved or alias symbols.
- **Get transcripts** and **exon info** obtain detailed information about available transcripts and exons based on the provided gene symbols.
- **Fusion check** determines if a genomic fusion event results in a frameshift of involved proteins.
- **Protein sequence** and **genomic sequence** enable the querying and manipulation of sequences in specific ranges or based on gene symbols.

In the following some key features of SeqCAT’s are highlighted through several use cases and scenarios. In addition, SeqCAT offers a variety of information endpoints from other resources, including amino acid structure and biochemical properties, reverse complementary transcripts, and pathway visualization based on data from KEGG (12).

### Use case 1: frontend application for everyday Wet-Lab work

In a study investigating T-cell lymphoma patients (14), genetic alteration data was collected from various sequencing methods, including whole-genome sequencing, whole-exon sequencing, and RNA sequencing. To integrate information from different formats, variants were annotated at both the protein and DNA levels. The information provided includes either the gene name and amino acid change or the changes in the nucleotide sequence at specific chromosomal locations.

**Figure 2.**
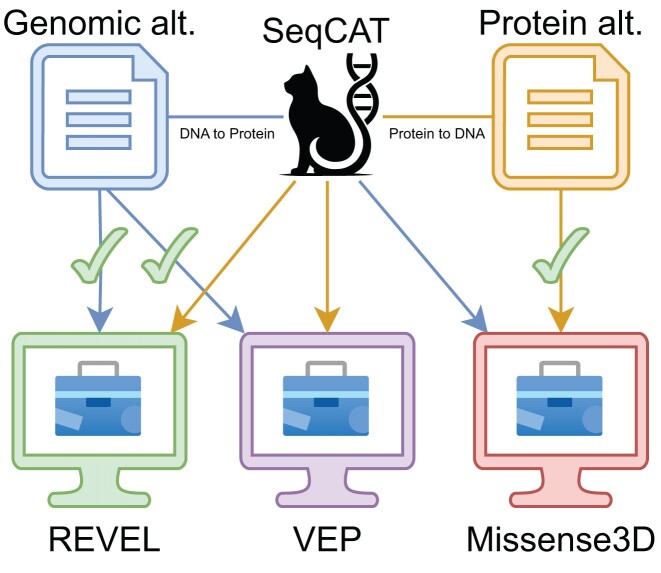
SeqCAT’s protein to DNA conversion and DNA to protein conversion. REVEL and VEP require the genomic position and nucleotide change (blue file), while Missense3D expects a protein change (orange file) to work correctly (green check marks). If the input file is not in the expected format, it requires prior conversion. SeqCAT is able to convert both files by interpreting protein changes as possible genomic changes (protein to DNA) and vice versa (DNA to protein). This allows for conversion of divergent files and makes them usable with established tools.

Standardizing diverse data sets is essential for using specialized annotation tools that require data in a specific format. For instance, the Variant Effect Predictor (VEP) (15) and REVEL (16) require both the genomic location and the nucleotide change to predict the impact of a variant on a protein, while Missense3D (17) needs details of the amino acid changes (see also Figure 2).

Such tasks can be easily solved with the SeqCAT’s web interface, which provides easy access to a variety of functions and resources, making it very useful in the laboratory or clinic. The ’Toolbox’ page provides several distinct tools at one place with a shared syntax for input and output. In this use case the protein to DNA conversion (Figure 2) feature was utilized to transform all protein-level variants into all possible corresponding DNA-level variants (e.g. *BRAF*: *V*600 → *chr*7 : 140753336*A* > *T*). Genomic nucleotide substitutions were mapped to their corresponding protein amino acid substitutions with DNA to protein conversion. To ensure that all gene names were current, converting any aliases or outdated names to their presently accepted symbols the gene name normalizer was applied (e.g. alias symbol: HGFR → approved symbol: MET).

### Use case 2: API for large data projects and automated workflows

The tasks described in use case 1 can also be performed without manual interaction with the web interface by switching to the SeqCAT API. The API facilitates the integration of data processing into custom pipelines or workflows without requiring manual access to the frontend application. The SwaggerUI documentation specifies services, input, and output formats, providing a visual interface to explore endpoints with correct syntax and results format previews (see Figure 1B). Moreover, the documentation provides sample input to demonstrate single data- or batch input. Examples for erroneous inputs show the error codes of API in the output (e.g. endpoint: */GenomicToGene/*).

SeqCAT’s API is already integrated in VUS-Predict (18), MTB-Report (19), and the variant interpretation framework Onkopus. This exhibits the robustness and stability of SeqCAT in everyday use. The projects utilize protein to DNA conversion, DNA to protein conversion, liftover and FusionCheck to preprocess the data and prepare it for other variant interpretation tools like VEP, REVEL, AlphaMissense and ClinVar (15,16,20,21). Genomic fusions are annotated if the fusion is ’in-frame’ or ’out-of-frame’ and therefore shifts the open reading frame, most probably resulting in an inactive protein.

### Use case 3: fast retrieval and manipulation of biological sequences

SeqCAT provides a variety of tools for accessing and editing sequences at the chromosome, transcript, or protein level. The protein sequence function allows to obtain the wild type as well as the modification of protein sequences through substitutions, insertions, or deletions as defined by the HGVS (e.g. *EGFR:p.G796D*). The modified sequence can be copied or downloaded in the FASTA format and can be used as input for further analysis e. g. for 3D structure analysis using programs such as AlphaFold or OmegaFold (22,23). Manual counting to identify the altered amino acid in the wild type sequence is no longer necessary. Using HUGO gene names as input, SeqCAT will return the AA sequence based in the MANE Select transcript. It is also possible to retrieve sequences based on specific alternate transcripts using the corresponding NCBI transcript identifier.

Genomic events, such as breakpoints, copy number variations, and fusions, can be automatically evaluated by accessing genomic sequences using given start and stop positions. The DNA sequence feature supports this analysis, enabling researchers to efficiently interpret complex data by quickly retrieving such sequences and searching for specific patterns.

### Use case 4: local repository for secured networks

In environments where data protection and data privacy is crucial, such as clinics with strict security policies, internet access is often limited or unavailable and sensitive data should not be transferred to public networks. This presents a challenge for using libraries that depend on online databases, such as Ensembl’s API (24–26), through tools like the BioMaRt R-package (27). These situations typically require adjustments to network security systems to ensure safe access. In addition, entry to local data is much faster and independent of the availability and response time of the often heavily used external APIs.

SeqCAT can be locally installed and dockerized from the source code by using the provided docker files (8). In addition, pre-built docker images are available on the public Gitlab repository: https://gitlab.gwdg.de/MedBioinf/mtb/seqcat.

## Discussion and conclusion

The Sequence Conversion and Analysis Toolbox –SeqCAT– is a valuable tool for managing and analyzing sequence data. It originated from several projects dealing with sequence conversions. The initial goal was to implement and maintain the underlying functionality once and focus on the primary projects. SeqCAT has developed into a toolbox that provides expertise for sequence manipulation, such as identifying the use of indexes or positions and determining whether numbering starts at zero or one? What resources are available for different tasks? How are alterations described and what is the definition of a primary transcript? Or is the coding strand backwards or forwards?

SeqCAT is a versatile toolbox that has demonstrated its benefits in several projects. This has led to the idea of providing a user-friendly web interface to establish SeqCAT in everyday laboratory work. It can be used as the first resource for various tasks related to DNA or protein sequences and other sequence-related information. The user interface is modern and user-friendly, providing consistent handling across all functions. Users are relieved of the burden of remembering which web page provides a tool for a specific task and how to use it.

The two-sided approach of SeqCAT, the web interface and the API, also allows an easy migration from initial tests to large data projects in a kind of rapid prototyping. While developing a protocol, the user can use the web interface to manually explore data sets and develop a protocol. If the research approach is promising, it is a small step to implement some scripts with a pipeline that now includes the same SeqCAT functions from the API to process large data sets in a (semi-) automatic way. Here another advantage of SeqCAT can support the user. The required data to process a user query is kept locally, ensuring constant processing time that is independent of the availability and response time of external APIs. Additionally, a local installation provides data privacy until the final results are ready for publication.

As genetic research advances, tools like SeqCAT that offer ease of use and comprehensive functionality will be crucial in navigating the complexities of sequence data. The development of SeqCAT is a major advance in bioinformatics and sets the pace for future improvements in genetic analysis and data management.

## Data Availability

SeqCAT is free and open to all users under Creative Common License (CC BY-SA 4.0). It is designed with data privacy in mind. So no login is required. No data are transferred to third party partner, therefore no cookies are required. No user data is stored on the servers. The website is available at https://mtb.bioinf.med.uni-goettingen.de/SeqCAT/.
